# The roles of gene duplications in the dynamics of evolutionary conflicts

**DOI:** 10.1098/rspb.2024.0555

**Published:** 2024-06-12

**Authors:** María del Pilar Castellanos, Chathuri Devmika Wickramasinghe, Esther Betrán

**Affiliations:** ^1^ Department of Biology, University of Texas at Arlington, Arlington, TX 76019, USA

**Keywords:** gene duplication, evolutionary conflicts, sexual conflict, arms race, conflict resolution

## Abstract

Evolutionary conflicts occur when there is antagonistic selection between different individuals of the same or different species, life stages or between levels of biological organization. Remarkably, conflicts can occur within species or within genomes. In the dynamics of evolutionary conflicts, gene duplications can play a major role because they can bring very specific changes to the genome: changes in protein dose, the generation of novel paralogues with different functions or expression patterns or the evolution of small antisense RNAs. As we describe here, by having those effects, gene duplication might spark evolutionary conflict or fuel arms race dynamics that takes place during conflicts. Interestingly, gene duplication can also contribute to the resolution of a within-locus evolutionary conflict by partitioning the functions of the gene that is under an evolutionary trade-off. In this review, we focus on intraspecific conflicts, including sexual conflict and illustrate the various roles of gene duplications with a compilation of examples. These examples reveal the level of complexity and the differences in the patterns of gene duplications within genomes under different conflicts. These examples also reveal the gene ontologies involved in conflict and the genomic location of the elements of the conflict. The examples provide a blueprint for the direct study of these conflicts or the exploration of the presence of similar conflicts in other lineages.

## 1. Introduction

Since gene duplications were first described in genomes, scientists have been wondering about their role in genome evolution. For example, Ohno asked, ‘Why gene duplication?’ in his book in 1970 [1]. As genome sequences accumulate, we are still providing a detailed answer to this question and describing the reasons for duplicated genes to be retained in genomes [2,3]. In this review article, we describe the changes that gene duplications bring about in genomes and compile examples where gene duplicates contribute to the evolution of evolutionary conflicts.

## 2. Major sources of evolutionary conflict in the genomes

Evolutionary conflicts occur because of antagonistic selection between different individuals, life stages or levels of biological organization [4,5]. These conflicts have been recognized to exert persistent and strong selective pressures on organisms and their genomes [5].

**Figure 1.**
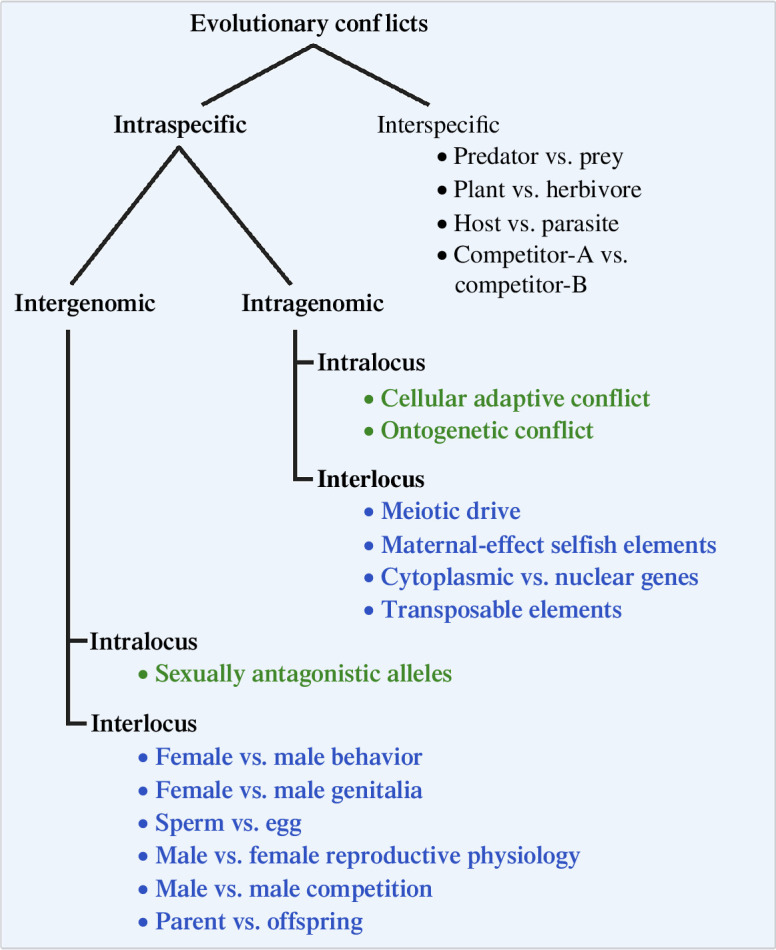
An overview of the main forms of evolutionary conflicts in organisms (modified from [5]). On the left in bold, we show the forms of evolutionary conflict we discuss, i.e. intraspecific conflicts. In blue, we show interlocus conflicts, i.e. the types of conflicts where gene duplication might spark or fuel conflict. In green, we show intralocus conflicts, i.e. the types of conflicts where there might be trade-offs resolved by gene duplication.

In figure 1, we redraw and expand a diagram depicting the major sources of evolutionary conflict after a publication by Rice [5]. There are three major categories across levels of biological organization by which we can classify evolutionary conflicts, whether they occur within or between species, within or between one individual or genome, and within or between one locus or multiple loci. Interspecific conflicts occur when there is predation, parasitism or competition and the individuals from the interacting species are under selection to adapt to the changes in the other species. These like other conflicts often persist and are the source of so-called red queen processes that are characterized by constant changes on both sides of the conflict to ‘stay in the same place’ [5]. Intraspecific conflicts can be intragenomic or intergenomic conflicts if they affect genes that reside in the same or different individuals of the same species, respectively, and intralocus or interlocus.

## 3. Features of gene duplications

**Figure 2.**
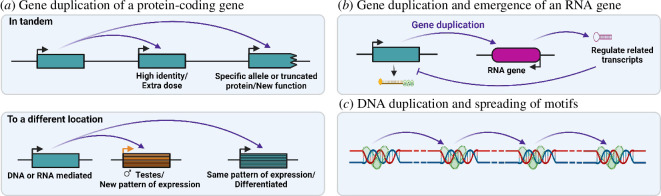
Gene duplication can have immediate effects on the genome. (*a*). If gene duplication occurs in tandem and there is no sequence divergence, it will have a dose effect, but if there are differences it can have functional consequences that build on the pre-existing function. If the gene duplication is not in tandem, it has higher chances of acquiring a different expression pattern and divergent function. (*b*). Partial gene duplications can evolve to produce small regulatory RNAs. (*c*). The duplication of DNA motifs can spread binding ability along a chromosome.

Whenever a gene or DNA region is duplicated, its function might duplicate and evolve new interactions that build upon pre-existing interactions (figure 2; [1,2]). Here, we highlight the features that confer upon them the potential to be effectors or modifiers of evolutionary conflicts. If a gene is duplicated and highly similar to the initial gene, it might have the effect of increasing the dose of the protein or RNA the gene encodes for. If, instead, it is a duplication of a particular allele or part of a gene it might immediately have new interactions or evolve to be an RNA gene and interfere with related transcripts. After duplication, non-coding antisense RNAs might evolve to affect transcript stability using the extant RNA complementarity. If the duplication is to a new location, the gene duplication might evolve a new pattern of expression or be able to diverge from the original copy because it will experience less gene conversion. This is often the case with retroposed copies of genes [6]. DNA-binding sites can also duplicate along a chromosome spreading particular interactions. We see these different types of gene duplications affecting the dynamics of evolutionary conflicts in different ways.

In the rest of the review, we provide examples of what we know about the role of gene duplications on intraspecific conflicts, including sexual conflict. We highlight that each type of intraspecific conflict appears to lead to specific genomic transformations involving gene duplications as it evolves.

## 4. Gene duplication might spark and/or fuel evolutionary conflict

**Figure 3.**
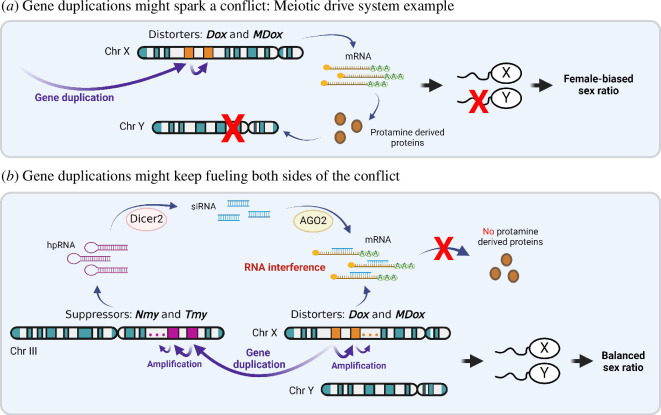
Illustration of how gene duplications provide the genetic information and pre-existing cellular interactions that can spark and continue to affect evolutionary conflicts. We use a well-known meiotic drive system as an example [7–11]. (*a*). Gene duplication creates two paralogous protein-coding genes. They are the X-linked drivers *Dox* and *MDox*. They are derived from protamines and likely interfere with protamine function and Y-bearing sperm leading to a female-biased sex ratio. (*b*). Suppression of *Dox*, *MDox* and other members of this gene family that are undergoing gene amplification involves RNA interference (RNAi) from paralogous RNA loci on an autosome that are also rapidly amplifying and diversifying and produce small interference RNAs (siRNAs). See main text for more details.

Gene duplications can initiate an evolutionary conflict or fuel the two sides of a conflict affecting its dynamics [4]. These are roles of gene duplications related to interlocus conflicts (shown in blue in figure 1). These roles evolve via one of two functions: (i) protein-coding gene duplication and/or modification of the existing protein interactions leading to or modifying the conflict or (ii) non-coding antisense RNAs affecting transcript stability using the extant RNA complementarity (figures 2 and 3).

### (a) Intraspecific intragenomic interlocus conflicts

Interlocus conflicts within a genome (i.e. intragenomic) occur because of the interactions between the host cells and selfish genetic elements or between organelles and the nuclear genome. We have good evidence of their existence and of gene duplications being often at the origin of the clashing interactions and/or providing fuel for one or both sides of the conflict in the arms race that takes place as those systems interact [12]. We illustrate those roles below with examples following the classification from figure 1.

Meiotic drive systems are selfish genetic systems that distort the faithful Mendelian chromosomal segregation in the male or female germline passing themselves more often to the next generation. In *Drosophila melanogaster* males, a well-known segregation distortion (*SD*) system involves a partial gene duplication (Sd-*RanGAP*) of a nuclear transport protein-coding gene, *RanGAP*, linked by inversions to a short satellite (*Responder*/*Rsp*) [13]. This combination is passed to the offspring most of the time in heterozygotes. The sperm with the wild-type chromosome, *SD^+^*, carries a long satellite (*Rsp^+^*) and fails to mature. It fails to transition from DNA interacting with histones to DNA interacting with protamines and to condense further. How a nuclear transport protein duplicate affects this transition is not well understood. It might involve either a direct interaction or the failure to transport enough small RNAs to silence the long satellite as Sd-RanGAP mislocalizes in the nucleus [13].

Male meiotic drive systems that affect sex ratio are called sex-ratio distortion systems. In the *Winters* system, a sex-ratio distortion system of *Drosophila simulans*, *Mother of Dox* (*MDox*) and *Distorter of X* (*Dox*), which originated from the duplication of *MDox* on the X chromosome, are drivers (figure 3*a*) [7,8]. They are passed to offspring more often, producing more females. *MDox* has a complex chimeric origin including parts derived from protamines and might be involved in the compaction of DNA in sperm [9]. *Dox* and *MDox* are members of a large, recently expanded gene family of likely protein-coding genes. The silencing of *Dox* and other members of its gene family has now been shown to involve RNAi and multiple paralogous small interfering RNA loci that are rapidly evolving, amplifying and diversifying by gene duplication [10,11]. This is likely an arms race between drivers and suppressors of the drive (figure 3*b*) [9]. The extremes in this process might be represented by the gene duplications, often in tandem, of genes on the X and Y chromosomes [14,15]. They might include some cryptic or old meiotic drive systems like *Stellate* (*Ste*) and *Suppressor of Stellate* (*Su(Ste*)) in *Drosophila* [16].

In *Drosophila*, expansions and contractions of the number of genes, and fast protein evolution of protamines themselves or DNA- or RNA-mediated duplication of nuclear transport genes that have evolved male germline-specific expression have been proposed to be associated with meiotic drive systems [17–21]. Duplicates of centromeric histone proteins have been proposed to be suppressors of centromere drive, a meiotic drive system where centromere quality determines how often that chromosome ends up in the oocyte biasing Mendelian chromosomal ratios in females [22].

The evolution by gene duplication of a maternally deposited toxin and antidote combinations that can exhibit selfish transmission has also been observed. For example, the maternal-effect toxin, *sup-35*, in *Caenorhabditis elegans* is a duplication of the gene *rmd-2* [23] and is suppressed by *pha-1*, a gene of unknown origin. Because this is a system that is fixed, i.e. present in all the individuals of the population, worms inherit *sup-35* and, if they do not have *pha-1*, they die from a pharyngeal defect. This led researchers to initially classify *pha-1* as essential for pharynx development.

Transposable elements (TEs) are also selfish elements. They are nucleic acid sequences that horizontally enter genomes and copy themselves in the germline to pass vertically to the next generation. This incurs costs for the host and introduces interlocus intragenomic conflict in the genome (figure 1). TE suppression can involve the evolution and diversification of gene families with roles, for example, in the regulation of TE transcription or restriction by RNA editing [24–26]. For example, Kruppel-associated box zinc-finger proteins (KRAB-ZFPs) are a very large family of transcription factors, with most members recently described to be involved in the recognition and silencing of TEs [25]. These gene families evolve rapidly, under positive selection, and experience turnover (duplication and loss) that is best explained by an arms race model where a new duplicate increases in frequency because it represses a kind of TE but can later be lost as the element degenerates [25].

Organelles and nuclear genomes might also be in conflict within an individual, and uniparental transmission of organelles is inferred to have evolved to suppress selfish organelles [27]. For example, mitochondria are only maternally transmitted and their genome has been proposed to be feminized. This might contribute to explaining why male-specific nuclearly encoded mitochondria gene duplicates have evolved specific mitochondria functions in the male germline [28,29].

The systems introduced above increase complexity in the genomes, experience high turnover and fast evolution and operate in different ways in the male and female germlines. The increase in complexity is sometimes ‘unnecessary’ for an individual’s fitness, as is the case of the *C. elegans* toxin and antidote that can be deleted from the genome without any deleterious effect [23].

### (b) Intraspecific intergenomic interlocus conflicts

Interlocus conflict between different individuals of a species (i.e. interlocus intergenomic conflict) can occur (figure 1), and gene duplication can again provide fuel for one or both sides of the conflict as those individuals interact [4]. These interactions can be between sexes, between different individuals of one sex that engage in offence/defence tactics or between parents and offspring [5].

Sexual conflict occurs because sexes have distinct reproductive roles. One form of sexual conflict is interlocus sexual antagonism, which involves the interactions between male and female loci [5].

The interactions and evolution of proteins transferred during internal fertilization provide some well-supported examples of interlocus sexual antagonism. In *Drosophila*, male accessory glands produce seminal proteins that are transferred to the female with the sperm during mating. These are known to evolve fast and under positive selection as they are proposed to be affected by male–male competition and male–female antagonistic interactions [30]. These proteins in flies have been characterized in several species [31–33], and lineage-specific gene duplications and diversification are observed [32]. A well-known seminal protein is the sex peptide (SP). SP stimulates egg production, reduces receptivity of the female and promotes sperm release from storage benefiting the male, but it has been observed to incur a female cost on viability or lower remating rate [34–36]. SP has been under strong selection [37], and this is also observed for other seminal fluid proteins with related functions in flies [34]. In females, reproductive proteins appear to coevolve with accessory gland proteins [35]. Female reproductive proteases have been described to be tandemly duplicated, showing population-specific patterns of gene turnover and evolving under positive selection and gene conversion supporting their coevolution under interlocus sexual antagonism with a role in the degradation of seminal fluid proteins [38,39].

Even during fertilization, which is essential for reproduction, male and female protein interactions are inferred to be antagonistic because males are competing to fertilize as many eggs as possible but females are under selection to prevent polyspermy [40]. Gene turnover and fast protein evolution under strong and changing selective pressures for genes involved in fertilization have been observed in vertebrates as well [41]. The function of duplicated proteins in fertilization has been reviewed recently [40] and uncovered duplicated genes in males and females with constantly changing interactions.

The genetic basis of male versus male competition might also involve the fast evolution of gene families under selection as offence and defence male functions evolve [5]. This could take the form of sperm competition or competition for mates. The data support rapid functional changes in a tandemly duplicated gene family of a recently evolved chimeric gene (*Sdic*) with a function in sperm displacement [42,43], where strong selection and recent selective sweeps have been observed in *D. melanogaster* [44].

Parent versus offspring conflict over parental investment also involves both fast protein evolution and gene duplications in lineages where a placenta has evolved [45]. Parents express genes that provide resources to the offspring but not more than needed, but offspring might evolve to express genes that increase the amount of investment. Fast evolution and gene duplication of pregnancy-specific glycoproteins that are secreted by the mammalian embryo into the mother’s bloodstream and regulate the mother’s immune and vascular systems have been observed [46,47]. Other pregnancy-related gene families in mammals have also been duplicated [48]. In some fish lineages where a placenta has independently evolved, genes likely involved in parental resource investment and interactions between mother and offspring are duplicated and evolving under positive selection [49].

The current data support the hypothesis that gene duplications contribute to the origin and the rapid changes on one or both sides of intergenomic interlocus conflicts between different individuals of a species.

## 5. Gene duplication might resolve evolutionary conflict

Intralocus conflicts (shown in green in figure 1) occur when alleles at a locus are maintained because they are selected for different and antagonistic functions, i.e. antagonistic pleiotropy. These conflicts lead to evolutionary trade-offs [4,50]. Gene duplication might facilitate the split of these functions and allow for the evolution of independent genetic bases for the two traits resolving the conflict at that locus [51].

### (a) Intraspecific intragenomic intralocus conflict

Intragenomic intralocus conflict occurs when alleles at a locus are selected for different antagonistic functions within an individual. They can come in the form of benefits from overdominance [1], protein adaptive conflict [52,53], cellular adaptive conflict or ontogenetic conflict [54,55]. The need for both alleles can be fulfilled by gene duplication [1]. For example, an anthocyanin pathway gene, *dihydroflavonol-4-reductas*e, has been duplicated twice in plants. The data support the hypothesis that the protein was performing several functions that have been optimized under positive selection after gene duplication revealing the existence of an adaptive conflict resolution [52,53]. We rarely have evidence that allelic variation was present before gene duplication occurred, but there is strong theoretical and empirical support for the process of resolving intralocus antagonism by the fixation of gene duplications [51–53].

### (b) Intraspecific intergenomic intralocus conflict

Intergenomic intralocus conflict occurs when alleles at a locus are selected for different antagonistic functions between individuals of a single species. One such example is intralocus sexual antagonism, i.e. when alleles at a locus have antagonistic effects in males versus females. The existence of intralocus sexual antagonism has been well documented from negatively genetically correlated effects between sexes: negative effects in fitness in one sex after selecting for the other sex, direct measures of the effect of clonal genomes on both sexes and antagonistic effects of alleles during development in both sexes [56–60].

**Figure 4.**
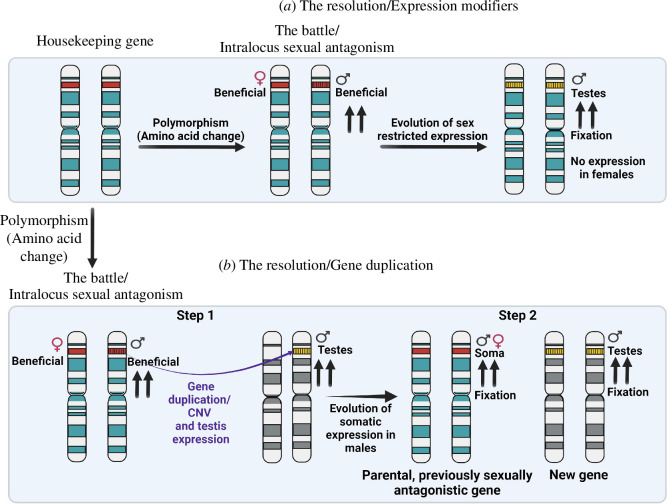
Illustration of a mutation producing an allele/amino acid change (shown as vertical lines in the housekeeping gene shown in red) with antagonistic effects in males and females leading to intralocus sexual antagonism, i.e. a battle for a different allele by either sex and its resolution [61]. The two vertical arrows pointing up point to the increase in the frequency of the particular chromosome under positive selection exerted by the male or both sexes. (*a*). Illustration of intralocus sexual antagonism resolved by the evolution of modifiers that restrict or bias expression (shown in yellow) to one sex. (*b*). Intralocus sexual antagonism can be resolved by gene duplication and the evolution of male germline-specific expression as this is the tissue exerting strong sexually antagonistic selection. We might end up with a male-specific gene and a gene expressed in females and male soma. The parental gene was under intralocus sexual antagonism, but the new male-biased gene might never have been under intralocus sexual antagonism. We rarely have data on all the steps of these models, but the number of male-specific paralogues in the genomes supports the second model of resolution. In model B, we do not have examples of male-specific copy number variants (CNVs) segregating and increasing in frequency. Note that many steps will take place under positive selection.

Gene duplication coupled with the evolution of sex-biased gene expression (figures 2*b* and 4*b*) might resolve intralocus sexual antagonism and contribute to the evolution of sexual dimorphism. Support for this process comes from strong male germline-biased expression and specific regulation of many duplicated genes, the abundance of some gene duplications, and strong selective pressures that lead to recurrent duplication and fast evolution under positive selection of some duplicates. We review these features below.

Gene duplications often have male germline-biased or male germline-specific expression [62–70] and are often duplicated by means of retrotransposition [6,69,71–73] in insects and mammals. There is evidence that duplicated gene functions have repeatedly evolved tightly regulated expression and specialized function in the male germline [18,63,64,72,73]. The parental genes have been shown to be enriched for particular housekeeping functions including nuclear-encoded energy-related mitochondria genes in flies [66], glycolysis genes in mammals [74,75], transcription factors [76,77], ribosomal function [77,78], nuclear transport [19,21,79], proteasome subunits [80] or centromeric histones [22]. This reveals a functional role of these duplications independently of the known broad and potentially spurious expression of the genome in testes during histone-to-protamine transition [81] and independently of meiotic sex chromosome inactivation (MSCI; see below). This is because many of these genes are autosome to autosome duplications that have evolved highly male germline-specialized functions.

Duplicated nuclear genes enriched for energy-related mitochondrial function have also evolved and might be a way by which males can evolve mitochondrial function independently of females [66]. In mammals, male-specific glycolysis genes have originated through gene duplication and have the specialized function of providing energy for sperm motility [82]. It seems that strong selection for duplication and specialization of glycolysis genes or nuclear-encoded mitochondria genes has occurred in mammals and *Drosophila*, respectively.

It was recently revealed that specialized ribosomal function is also needed for sperm function in mammals and is attained by ribosomal protein duplicates [78]. The same is true for proteasome subunits in flies [80]. Transcription factors needed for male-specific expression of genes have evolved through gene duplication and specialization as well [76,77]. Some of these genes might have evolved because of intragenomic conflicts that are different between sexes fueling sexual conflicts.

The resolution of intralocus sexual conflict might often involve gene duplication [58,66,83]. The data introduced above support this hypothesis. Under such a scenario, we expect to observe the evolution of a strongly male-biased, often male-germline-specific, gene duplicate and a gene expressed in female and somatic cells from an initially housekeeping gene [65,66,83]. We proposed a detailed model that involves the role of positive selection at every step in fixing these new male-biased gene duplications (figure 4; [61,66]). Importantly, under this model, sex-biased gene expression does not need to be a footprint of past intralocus sexual antagonism at that locus as often argued in the past [84–87]. If sex-specific gene expression is observed after gene duplication, this can correspond to a sex-biased gene that is a duplicate of a parental gene that was under intralocus sexual antagonism but was never itself under intralocus sexual antagonism [61]. Again, as mentioned above, the evidence of antagonistic alleles existing before the gene duplication (i.e. step 1 in the model; figure 4*b*) might be lost, and we discuss the existing direct support for this model in §7.

A detailed population genetics model where a male-biased gene and a female-biased gene evolve from an initially broadly expressed gene under intralocus sexual antagonism has been developed providing details about the genetic and population parameters that might affect the process [88]. This model also applies to the evolution of sex-specific alternative splicing after domain duplication, another role of gene duplication in the resolution of intralocus sexual antagonism related to specific domain functions [83]. We again do not have evidence that allelic variation was present before domain duplication occurred, but multiple instances of sex-specific alternative splicing support this process [89,90].

While strongly sex-biased genes might harbour less standing sexually antagonistic variation and be part of sexual conflict resolution (figure 4*a*,*b*) [50,87], it is relevant to consider a particular instance in *D*. *melanogaster* where a gene ancestrally expressed in both sexes duplicated and subsequently evolved into a male-specific gene and a female-specific gene with essential roles in spermatogenesis and oogenesis, respectively [91]. The genes are named *Apollo* and *Artemis*. They were described to have evolved to split the ancestral function between sexes, likely under intralocus sexually antagonistic pressures. It is, however, relevant to highlight that the loss of *Apollo*, a male-specific gene, is still beneficial for females, and the loss of *Artemis*, a female-specific gene, is still beneficial for males revealing that the sex-specificity of the sex-specialized gene might either not be complete, i.e. there is leaky expression, or that having that sex specificity is costly. This example supports the hypothesis that the complete resolution of intralocus sexual antagonism is challenging to achieve and that even sex-specific genes that evolved by means of gene duplication might have a cost in the other sex [91–94].

## 6. Evolutionary conflicts drive the evolution of sexual dimorphism and sex chromosomes

Male and female genomes often contain (nearly) the same genetic material. However, as mentioned above, the two sexes often have different optimal fitness strategies for reproduction, experience different selective pressures and interact antagonistically with each other, resulting in sexual conflicts [57]. The degree of sexual dimorphism in a species might depend on the level of sexually antagonistic interspecies and intraspecies interactions accentuated by any germline sex-specific intragenomic conflict differences as mentioned above. Selective pressures in the two sexes and their interactions might contribute to the fast evolution and diversification of genes under interlocus sexual antagonism. In addition, continuous cycles of intralocus sexual antagonism and resolution by the evolution of sex-biased genes either by regulatory changes or gene duplication plus regulatory changes may generate diversification (figures 2 and 4). For example, the evolution of testis-specific regulatory networks has occurred through gene duplication of regulatory proteins that control testis-specific genes in flies [76].

**Figure 5.**
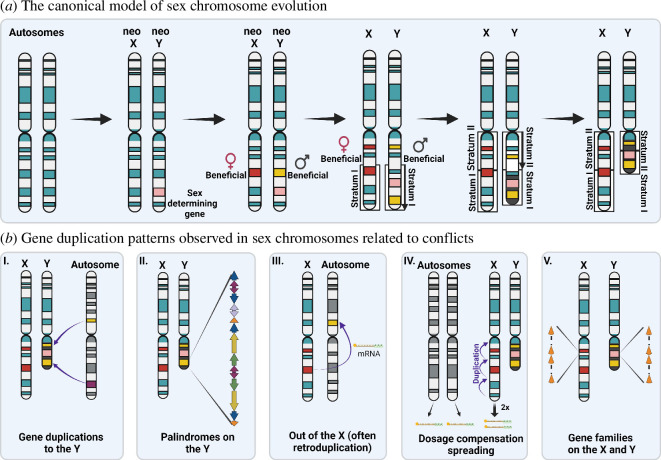
(*a*). The canonical model of the evolution of sex chromosomes: evolution of a sex-determining system, evolution of linkage with alleles with sex-specific effects and degeneration and divergence in steps/strata due to the lack of recombination. (*b*). Gene duplications are often observed to contribute to sex chromosome evolution that is likely driven by evolutionary conflict: (I) male-specific genes are gained on the Y chromosome, (II) palindromes evolve on the Y chromosome, (III) out of the X duplication pattern of male-specific genes is observed, (IV) dosage compensation on the X evolves by duplication/spreading of dosage compensation sequences and (V) the evolution of the same gene families on the X and the Y occurs. See the text for details and a discussion of those duplication patterns, the diversity of selective pressures related to intraspecific conflicts that might affect them and deviations from these canonical steps.

Sex chromosomes might evolve, in addition to sexual dimorphism, and contribute to accentuating differences between sexes and the evolutionary conflicts they experience. Several steps have been observed to recurrently take place in both male heterogametic and female heterogametic systems [84,95–98]. Figure 5*a* describes the canonical model of sex chromosome evolution focusing on an XX/XY system. Although many species lack distinct sex chromosomes [99], we focus on the recurring themes observed across the sex chromosome literature and their relation to evolutionary conflicts.

Sex chromosomes evolve from autosomes when recombination ceases at once or in steps/strata after the evolution of a sex-determining system. It is often assumed that this occurs because a locus where alternative alleles are favoured in each sex (i.e. intralocus sexually antagonistic selection) exists close to the sex-determining gene. Other hypotheses for why recombination ceases have also been suggested, including the linkage to a meiotic drive system [99]. The lack of recombination leads to the degeneration of the non-recombining region and the accumulation of TE insertions [100]. The non-recombining chromosome loses genes and dosage compensation evolves to maintain the expression level of genes in the heterogametic sex [101]. In addition, the non-degenerating sex chromosome might undergo MSCI in the germline of the heterogametic sex as unpaired chromosomes are known to condense during meiosis [102,103].

Gene duplications have been observed to play a role in sex chromosome evolution at almost every step and are related to evolutionary conflicts associated with the sex chromosomes (figure 5*b*). As the Y chromosome evolves to retain a few genes that acquire male-specific expression and function [84,96–98], it might be a favourable, i.e. not sexually antagonistic, location for male-specific genes and the Y may continue to gain male-specific genes that move by transposition or retrotransposition (figure 5*b* (i)) [91,98,104–106]. In addition, some of the male-specific genes on the Y duplicate and form palindromes (figure 5*b* (ii)). This arrangement might allow these genes to continue evolving fast under conflicts without degenerating [107–109]. An excess of housekeeping genes has been observed to be duplicated from the X chromosome to the autosomes, i.e. out of the X duplication pattern, and to acquire male germline function (figure 5*b* (iii)) [67,75,110–112]. It has been proposed that MSCI might be the reason for this pattern [75,110]. However, the fact that not all housekeeping genes on the X are duplicated but only some that acquire specialized function for the male germline strongly supports the role of intralocus sexual antagonism resolution as the basis for this pattern [21,61,66,79]. Duplication plays a role in the spread of the dosage compensation marks on the X chromosome as the Y chromosome degenerates (figure 5*b* (iv)) and has been described to be mediated by TEs [101,113]. Some gene families have been acquired by and amplified on the X and Y chromosomes (figure 5*b* (v)). Above, we mentioned the fact that these might be part of male germline sex ratio distortion systems and suppressors [14,15,114]. There is support for this hypothesis as there are gene families on both chromosomes (i.e. *Sly* and *Slx* and *Slxl1*) that have been shown to have an antagonistic role in postmeiotic expression and silencing of themselves and each other [115]. As sex chromosomes evolve under strong evolutionary conflicts, the genome changes dynamically, with gene duplications contributing to sex chromosome differentiation.

The fact that many Y-linked genes are testis specific in expression supports the role of sexual conflict. It would, however, be good to have direct support for all the hypothesized steps of the models. Theoretically, we expect intralocus sexually antagonistic selection close to the sex-determining region to produce divergence between the X and the Y in the recombining region [116] and this has been observed for sex chromosomes in *Silene latifolia* [117] and stickleback sex chromosomes [118]. However, the effects of the associated variation are unknown or alternative explanations for the X–Y differentiation cannot be ruled out [119]. This reveals that additional data are needed to have direct support for the selective pressures underlying the evolution of sex chromosomes.

## 7. What have we learned from copy number variants?

Population genomics, i.e. the sequencing of multiple genomes per species, has allowed the characterization of copy number variants (CNVs), i.e. the presence of gene families with different numbers of genes in different individuals’ genomes. CNVs might be observed due to gene duplications that are not fixed or due to gene losses in some of the genomes [120]. Recently, duplicated CNVs provide information about mutational gene duplication patterns and about the selective pressures operating on them as they increase in frequency [2,121].

CNVs can provide direct evidence supporting some of the proposed models of evolutionary conflict. Here, the model for the resolution of intralocus sexual antagonism by gene duplication is of particular interest (figure 4) [66,94]. However, we still lack evidence supporting all the different steps in this model, i.e. we have not identified any population where a new still segregating male beneficial allele duplicate (i.e. a CNV) has acquired male-germline expression (figure 4*b*; step 1) as intralocus sexually antagonistic variation is lost in the parental gene and the new gene fixes in the population (figure 4*b*; step 2). The current data on polymorphic CNVs supports that they are selected to increase the dose of a gene product [122]. In some instances, authors have seen recurrent duplication and consistent amino acid changes for new testis-specific genes from populations where variation exists for the parental gene at the protein level [123]. However, we do not know the fitness effects of every allele and duplicate to understand how well the model is supported. We do not know if the initially duplicated allele was the male beneficial allele (figure 4*b*; step 1) or if the initial effect was a dose effect.

With respect to some of the patterns of gene duplication in the evolution of sex chromosomes, RNA-mediated CNVs have confirmed, for example, that the ‘out of the X’ pattern of gene duplication is a selective pattern because the initial/low frequency/segregating duplications do not show it [124–126]. The Y chromosomes in seed beetles were revealed to harbour copies of the *TOR* gene that appear to contribute to increased sexual dimorphism and sexual conflict resolution [127]. Despite the difficulty of obtaining CNV data, it can be very informative about mutation and selection patterns.

## 8. Big picture and the road forward

There are many examples of gene duplications that have a role in the dynamics of evolutionary conflicts. These duplications provide a catalogue of patterns (table 1): fast evolution, high turnover, expanding and diversifying protein-coding gene families in the sex chromosomes and autosomes, expanding and diversifying small RNA gene families, translocated copies of house-keeping genes with sex-biased expression and particular gene ontologies, sometimes the same on both sides of the interlocus conflict, that depend on the type of conflict. While this catalogue is incomplete or in some instances surprising, e.g. it is not easy to infer what is going on until the whole system is described (i.e. toxin and antidote system in *C. elegans*), it serves as a guide going forward by providing the features to look for in the genomes that might be related to evolutionary conflicts (e.g. [10,11]) and to understand the underlying selective forces that drive genome evolution and lead to the genome complexities we observe. Nowadays, even repetitive regions can be manipulated, and the effects studied in model and non-model organisms [11,43] and more of those experiments are needed to gather additional support for some of the hypothesized effects.

**Table 1.** Summary of examples of gene duplications with a shown or supported role in intraspecific evolutionary conflicts.

Evolutionary conflict		Elements of the conflict	Type of gene duplication	Location	Function	Ref.
**Interlocus**	**Meiotic drive**	Drivers	**Fast-evolving** and often **high-turnover** protein-coding and RNA gene families **in tandem**	**Elements linked** and on the same chromosome, **including sex chromosomes**	**DNA condensation broadly** (nuclear transport, protamines…) and **centromere**	[7–11,13–22]
		Suppressors	**Fast-evolving** and often **high-turnover** protein-coding and RNA gene families **in tandem**	Elements on a **different chromosome** from drivers, **including sex chromosomes**	**DNA condensation broadly** (nuclear transport, sRNAs…) and **centromere**	[7–11,13–22]
	**Transposable elements**	TEs and their control	**Fast-evolving** and **high-turnover** gene families controlling TEs	Diverse location	**DNA condensation** and **RNA editing**	[24–26]
	**Organelle versus nuclear genes**	Feminized organelles versus male functions	Single gene evolving a **male function**	Different chromosome than original gene	**Energy** and/or **organelle** related	[28,29]
	**Male versus female**	Male versus female interacting genes	**Fast-evolving** and **high-turnover** gene families of sexually interacting genes	Different locations in the genome **including sex chromosomes**	**Reproduction** and behaviour	[31–40]
	**Male versus male**	Male versus male competition	**Fast-evolving** and **high-turnover** gene families of offence/defence genes	Single chromosome	Offence/defence **reproduction**	[42–44]
	**Parent versus offspring**	Parental investment in the offspring	**Fast-evolving** and **high-turnover** gene families of resource investment genes	Diverse location	**Placenta** and **bloodstream**	[45–49]
**Intralocus**	**Cellular adaptive conflict**	Alleles within a locus	**Single gene specializing** for an adaptive function	Same or different chromosome	Diverse	[52–55]
	**Sexually antagonistic alleles**	Sexually antagonistic alleles within a locus	Single **gene evolving a male- or female-specific** function	Often **different chromosome** than original gene	Diverse/**sexually antagonistic**	[66–79]

*Note:* Relevant recurrent features of evolutionary conflicts are highlighted in bold.

It has also become clear that, given the repetitive nature of some of the regions involved in a conflict, assemblies based on long-read data are needed to be confident about the inferences. As researchers sequence genomes and obtain comparative and population genomic data, the regions that are quickly changing, including duplicated genes [128] will help identify functions under selection and genetic variation segregating under conflict.

Instances of CNVs and parental gene allelic variation should be studied to understand the antagonistic selective pressures for new duplicates that increase in frequency. As mentioned above, CNV data is still needed to have a complete picture of how often a new allele or an increase in dose might contribute to conflict resolution.

With respect to the genomic location of duplicated genes involved in conflicts, the sex chromosomes have been observed to be front and centre of many of the dramatic changes occurring under conflicts. So, both young and old sex chromosomes should be studied, compared and manipulated [42,43,129] to understand all the steps and selective pressures they are undergoing in more detail. For example, when amplified X and Y genes are observed, it is imperative to understand where they appear first and with what effects. The Y chromosome accumulates TEs and can be a hot spot for ectopic recombination. This means that gene duplicates might appear that are deleterious and silencing of those might evolve on the X chromosome. So, it does not need to follow that amplified X and Y genes are X-linked selfish sex ratio distorters and Y-linked suppressors, and more data and experiments need to be performed to understand how often conflict occurs.

In addition, given how strong the selective pressures are related to evolutionary conflicts, genomes experience intense makeovers in a short time. The expectation is that as more data accumulates, more support will continue to emerge for species incompatibilities that might have evolved between species under evolutionary conflicts [5,130,131].

## Data Availability

This article has no additional data.
